# Arthroscopic treatment of chronically painful calcific tendinitis of the rectus femoris

**DOI:** 10.1186/2047-783X-18-49

**Published:** 2013-11-23

**Authors:** Xu Peng, Yong Feng, Guangxing Chen, Liu Yang

**Affiliations:** 1 Center of Joint Surgery, Southwest Hospital, The Third Military Medical University, Chongqing 400038, China

**Keywords:** Calcific tendinitis, Arthroscopy, Hip

## Abstract

**Background:**

Relatively large calcific tendinitis with persistent symptoms after extended periods of conservative treatment is an indication for operative therapy. Arthroscopy, as a treatment for calcific tendinitis of the hip abductors and calcinosis circumscripta, has been described previously; however, to our knowledge, the clinical and radiological response to arthroscopic removal of calcific tendinitis of the rectus femoris tendon has not.

**Methods:**

We present arthroscopic treatment of unusual calcific tendonitis of the origin of the rectus femoris and associated intra-articular lesions in 3 patients with chronic coxa pain.

**Results:**

Our cases show that hip arthroscopy is an effective therapeutic modality for calcific tendinitis of the hip joint with satisfactory short-term outcomes.

**Conclusions:**

Calcific tendinitis, although an uncommon clinical entity, should be a part of the differential diagnosis of acute or chronic hip pain.

## Background

Calcific tendinitis results from the deposition of calcium hydroxyapatite crystals in the periarticular muscle attachments [1]. It causes inflammation, necrosis, and loss of tissue structure. Its pathogenesis is unclear. It seems to be a multifactorial disease, in which traumatic [2], genetic [3], and local metabolic [4] factors are proposed in the etiology. The most common anatomic location of calcific tendinitis is the supraspinatus muscle [2] of the glenohumeral joint. The hip region [1] is also one of the frequently affected sites. However, calcific tendinitis at the site of origin of the rectus femoris muscle [5] is rarely encountered.

The differential diagnoses of calcifications in the rectus femoris include lumbar spinal disease, avascular necrosis, osteoarthritis, stress fractures, femoro-acetabular impingement, snapping hip syndrome, avulsion fractures of the rectus femoris, et cetera. An adequate history and physical examination are the first and the most important steps of the specific diagnosis. In cases of diagnostic uncertainty, advanced imaging techniques such as ultrasound, computer tomography (CT) or magnetic resonance imaging (MRI) should be used [6]. CT may be helpful in evaluating osseous involvement, and MRI can demonstrate soft-tissue edema. These methods can lower the misdiagnosis and allow the assessment of intra-articular lesions.

Surgical intervention with arthrocopy as a treatment for calcific tendinitis of the hip abductors [7] and calcinosis circumscripta [8] has been described before, but, to our knowledge, the clinical and radiological response to arthroscopic removal of calcific tendinitis of the rectus femoris tendon has not yet been. We present three cases of hip arthroscopic removal of calcific tendinitis within the origin of the rectus femoris tendon close to the acetabular rim.

## Methods

### Portal placement and calcium deposit location

**Figure 1:**
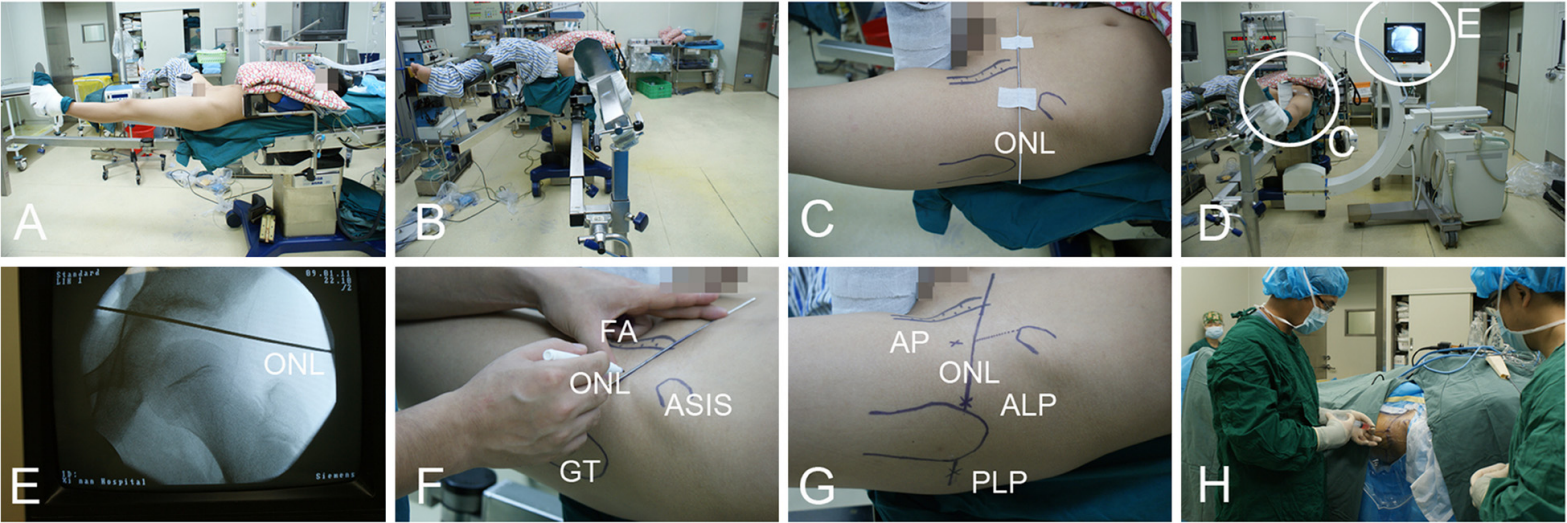
**Preparation and portal making. (A)** Place the patient supine on the traction table. **(B)** Position the hip to be operated on in extension, approximately neutral abduction, and 15° of internal rotation. **(C)** Landmarks outlined: femoral artery (FA); greater trochanter (GT); and anterior superior iliac spine (ASIS); optimal needling line (ONL). **(D)** Apply traction to the operative extremity, and confirm the correct position of the Kirschner wire as a metal marker for the optimal needling line. **(E)** Fluoroscopically, a vacuum sign, seen as a crescent-shaped area of radiolucency, appeared in the distracted affected hip and the Kirschner wire centered between the acetabulum-femoral head space. **(F)** The ONL was depicted along the Kirschner wire course on the skin with a marker pen. **(G)** Three modified portals were referenced from the optimal needling line - the anterior, anterolateral (AL), and posterolateral (PL) portals. **(H)** Establish the anterolateral portal first, and further distention is achieved by forcibly injecting 30 to 50 ml normal saline into the joint space.

The patient was placed on a traction table in the supine position (Figure 1A), with a C-arm image detector centered over the operative hip. Adequate hip distraction was performed to obtain approximately 10 mm of joint space (Figure 1B). Subsequently, a Kirschner wire was placed on the skin surface of the affected hip as a marker for the optimal needling line (Figure 1C). The radiograph in this projection attempted to locate the correct position of the Kirschner wire, which was placed between the acetabulum-femoral head space (Figure 1D,E). With this setup, the optimal needling line, an important skin surface landmark, was mapped along the Kirschner wire course on the skin with a marker pen (Figure 1F), which served as the reference line for all portal placements during hip arthroscopy. Three modified portals, anterior, anterolateral (AL), and posterolateral (PL) were established (Figure 1G-H). The anterior portal was made at the junction of a sagittal line drawn from the anterior superior iliac spine and the optimal needling line. The AL portal was established at the intersection of a line extended from the anterior border of the greater trochanter and the optimal needling line. The PL portal was positioned similarly to the AL portal except it was at the posterior margin of the greater trochanter. A standard diagnostic arthroscopy was first performed via the AL portal. Subsequently, exploration of the superolateral paralabral sulcus was made, allowing for an accurate and rapid intraoperative localization of the calcific materials, because usually the pathology presents with a calcific bulging sign in a superficial region and is large in size. When it is difficult to visualize any protuberance on the outer surface of the capsule side, intraoperative C-arm fluoroscopy may be valuable in localizing the deposits but this was not utilized in this series.

The Ethics Committee of the Southwest Hospital approved the study protocols, and all participants provided written, informed consent prior to participation in the study. The name of the file is ‘Arthroscopic treatment of chronically painful calcific tendinitis of the rectus femoris’. The number of the file is ‘KY201011’.

### Case reports

#### Case 1

A 45-year-old woman had presented with recurrent pain in her left hip for 2 years and that had become severe in intensity for 6 months. The dull pain was located around the groin area, not radiating to the lower back or the lower extremities. There was no previous trauma noted. In recent months, discomfort was induced by climbing a few stairs or walking for a long distance. The pain had not become sharp until 2 months before admission and conservative treatment with analgesics brought no relief.

Physical examination showed complete normal passive range of motion (ROM), which was substantially pain-free except in maximum flexion-internal rotation, negative impingement signs and Trendelenburg test, and no tenderness points around the bony landmarks. The Patrick’s (Fabere) test and resisted flexion-internal rotation of the hip were painful. Laboratory test results were normal.

**Figure 2:**
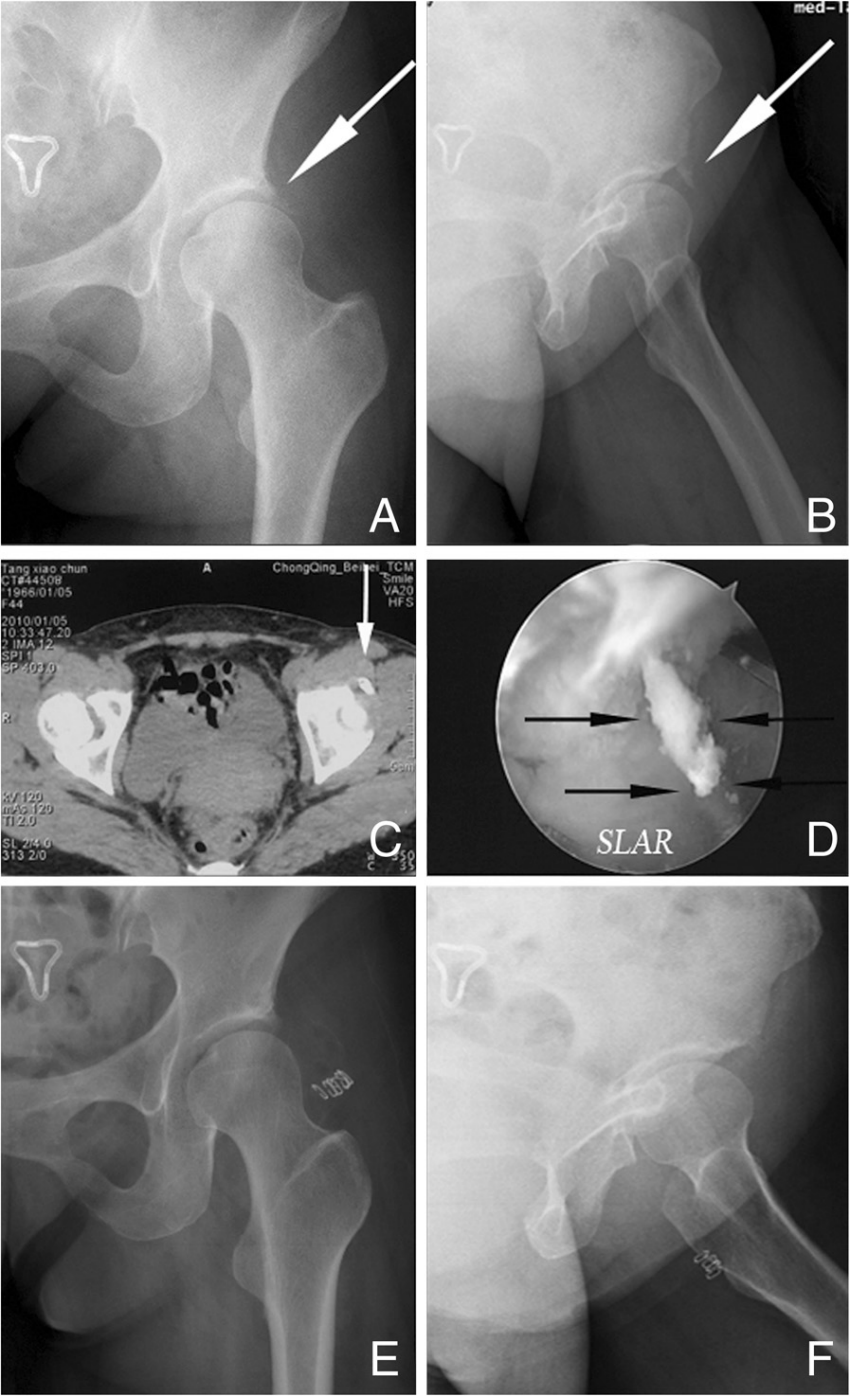
**Case 1 radiographic data. (A)** The anteroposterior (AP) view and **(B)** the frog-leg lateral view showed a well-formed triangular radio-opacity (arrow) just adjacent to the prominent acetabular roof. **(C)** The axial computer tomography slice through the superior part of the hip joint clearly demonstrated the variable calcifications (arrow) in the origin of the rectus femoris. **(D)** Arthroscopic view of toothpaste-like calcified material (marked with arrows; SLAR, superolateral aspect of the acetabular roof). **(E)** Postoperative AP and **(F)** lateral views presented no residual calcium shadow.

Anteroposterior- and lateral-projection radiographs of the left hip revealed a triangular homogeneous radio-opacity with well-formed borders just adjacent to the superior lip of the acetabulum (Figure 2A,B). This area corresponded to the tendinous origin of the rectus femoris. Subsequent CT scans confirmed the presence of calcification (Figure 2C).

The preoperative diagnosis was calcific tendinitis of the origin of the rectus femoris tendon. Because the conservative treatment was unsuccessful in alleviating symptoms, arthroscopic debridement was considered.

The surgical procedure started with an intra-articular diagnostic arthroscopy. With the aid of a sparing resection of the superficial capsule fibrous tissue, arthroscopy showed degeneration and calcific deposits at the origin of the reflected head of the rectus femoris tendon. With a longitudinal opening in line with the degenerated tendon course, white, soft, toothpaste-like material spurted out, which was then completely removed with blunt instruments (Figure 2D-F). During the procedure, a shaver was not systematically used so as to minimize the damage to the reflected head integrity and the possibility of tendon rupture. Intra-articular debridement was also performed. The specimen was submitted for pathologic examination, which confirmed the presence of hydroxyapatite crystals and chronic inflammatory infiltration.

#### Case 2

**Figure 3:**
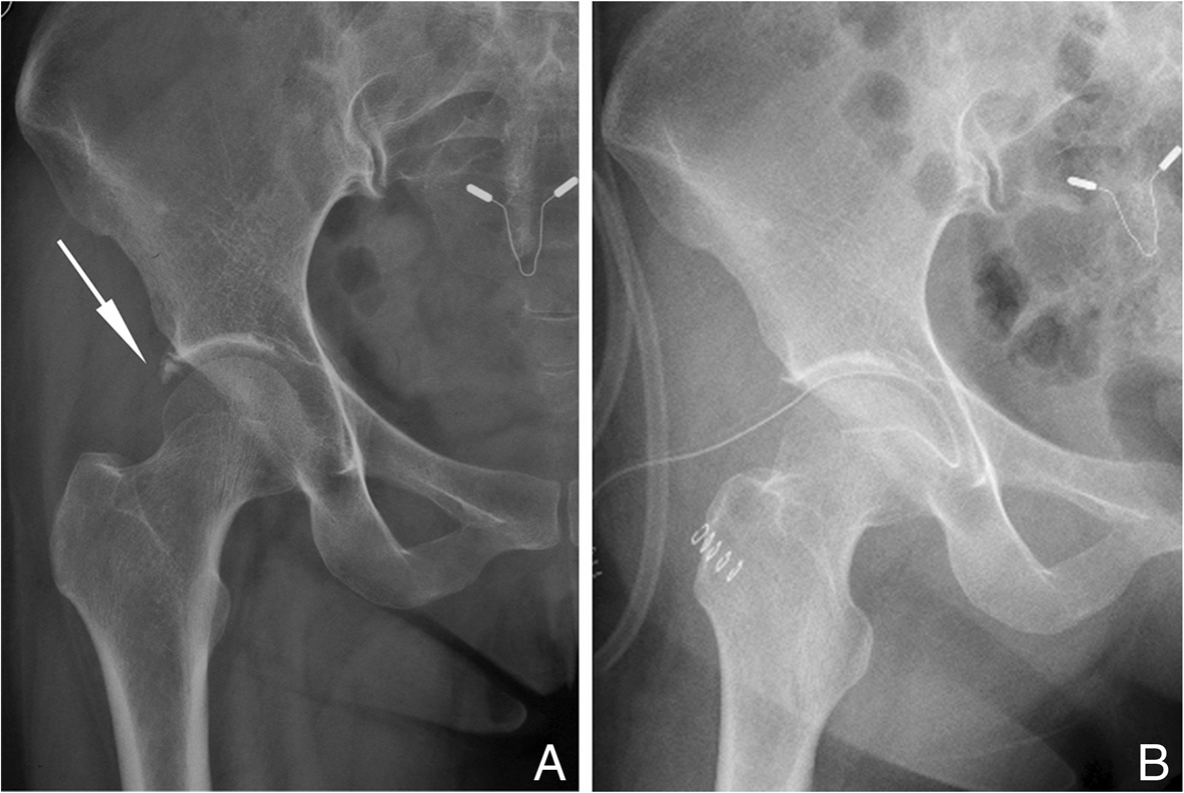
**Case 2 radiographic data. (A)** Preoperative radiographic anteroposterior view of the right hip with calcific densities located near the superior tip of the acetabulum (arrow) in case 2. **(B)** Radiographic anteroposterior view presented complete removal of calcification at postoperative day 1 in case 2.

A 38-year-old woman complained of repeated vague pain near the right lateral hip over the past 8 months. The last attack of pain, provoked by jogging, persisted for approximately 10 days but was not relieved with nonsteroidal anti-inflammatory drugs (NSAIDs). There was no history of previous injury and no fever, chills, or other systemic symptoms. The findings of musculoskeletal examinations were positive for pain in forced maximum flexion-internal rotation position of the right hip. Ely’s test and Patrick’s (Fabere) test were inconclusive. The rest of the examination as well as the general examination was not contributory. All laboratory findings were unremarkable. The radiograph of the pelvis revealed a linear-shaped calcium deposition in the lateral border of the acetabular rim (Figure 3A).

A preliminary diagnosis of chronic calcific tendonitis of the origin of the rectus femoris was made. After an appropriate discussion with the patient, arthroscopic surgical exploration with the intention to completely remove calcium deposits was undertaken. The same arthroscopic portals were established and an intra-articular diagnostic arthroscopy was performed as in the preceding case. Intra-operatively, milky fluid and a chalky deposit of calcium were debrided. Arthroscopic labral debridement was performed at the end of the surgical procedure.

#### Case 3

The patient was a moderately obese 55-year-old woman with complaints of right posterolateral hip pain after walking. Rest and analgesics had given no relief. She did not report any constitutional symptoms. The patient related her first sudden onset of hip pain to a twisting injury of the same hip 3 years earlier. Radiography of the hip joint performed at that time was reported to be normal. The injury was treated with rest for 3 weeks. Throughout the intervening years, the patient had chronic low-grade dull pains in the hip. Two months ago, the pain became acute and was exacerbated by hip motion.

Physical examination disclosed exquisite tenderness over the posterolateral area of the left hip joint, with the limitation of forced maximum flexion abduction of the right hip due to the pain. Patrick’s (Fabere) test was positive. Pain was elicited by moving the hip passively from FAbER (flexion-abduction-external rotation) position to EAddIR (extension-adduction-internal rotation) position. The rest of the physical examination was normal.

**Figure 4:**
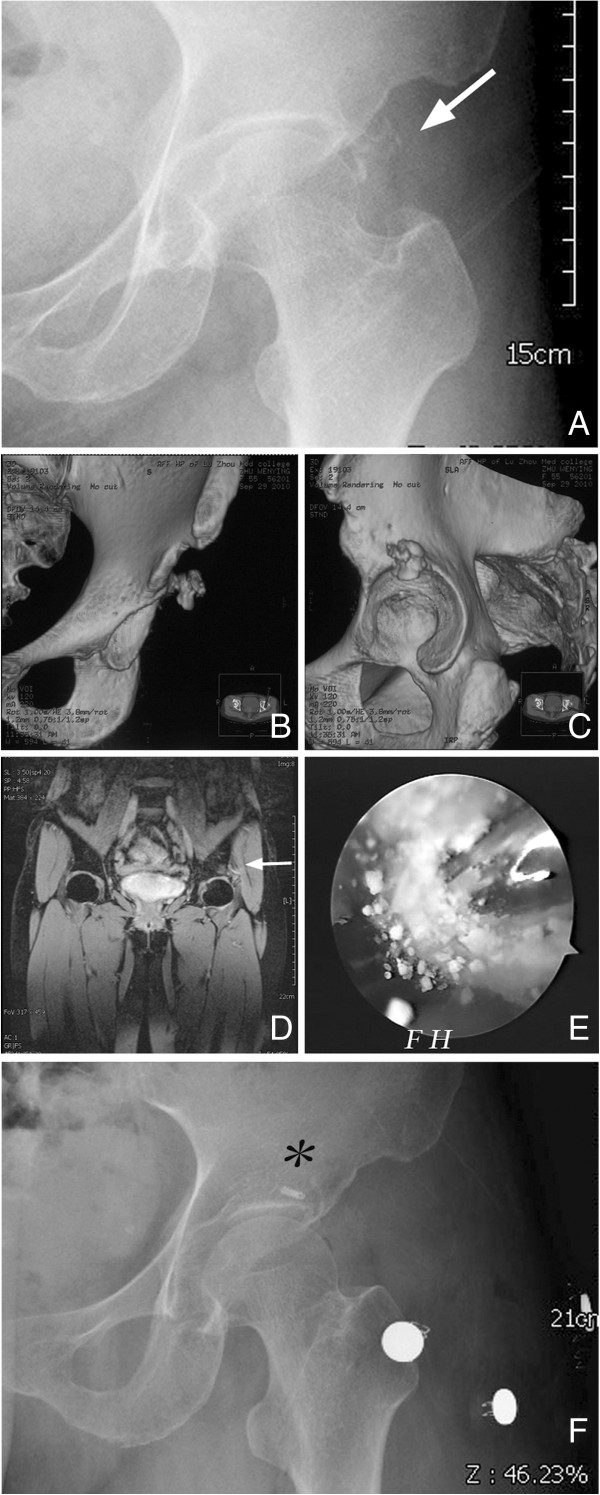
**Case 3 radiographic data. (A)** Anteroposterior (AP) projection of the left hip showing an ill-defined calcific spot (arrow) close to the acetabular roof. **(B)** Coronal and **(C)** sagittal three-dimensional reconstruction computer tomography images verified the large and irregular calcium mass. **(D)** The coronal enhanced fat-suppression phase arthro-MR slice showed miscellaneous signal changes of the origin of rectus femoris combined with labral tear (arrow). The tendon fibers had been displaced by the calcium mass and there was a large bone erosion of the acetabulum with peripheral soft-tissue edema. **(E)** Typical snowstorm-pattern after incision of the superficial capsular over the calcification (FH, femoral head). **(F)** Postoperative AP view demonstrating complete removal of calcific material; ^*^non-absorbable suture anchor for the labral repair.

An anteroposterior projection radiograph and CT of the left hip revealed a 1.2-cm diameter radio-opacity with irregular borders, located near the superior tip of the acetabulum (Figure 4A-C). MRI showed evidence of labral pathology and verified the calcification (Figure 4D).

The diagnosis of chronic calcific rectus femoris tendinitis of the left thigh was made and treatment with arthroscopic surgery was initiated. A similar arthroscopic technique was used for this patient as described previously. During the procedure, the calcium deposit was immediately apparent by simple arthroscopic inspection from the acetabulum side. After opening its capsule, the typical snowstorm pattern appeared (Figure 4E). The deposit was cleared out. In addition, a type I labral tear was verified during the hip arthroscopy. A non-absorbable suture anchor was used to stabilize the labrum tissue back to the rim of the acetabulum. A radiographic control was performed at the end of the procedure to evaluate the amount of deposit remaining (Figure 4F). Histological examination of the collected sample showed abundant calcium salt deposits surrounded by several mature trabecular bone and fibrous tissues.

## Results

Our cases show that hip arthroscopy is an effective therapeutic modality for calcific tendinitis of the hip joint with satisfactory short-term outcomes. Case 1 result: The pain subsided within a few days and immediate postoperative radiography revealed no residual calcification. Straight-leg raise exercises were started on the first day post surgery and resisted knee-extension strengthening exercises on postoperative week 6. There was intact function of the extensor mechanism and full strength of knee extension in contrast with the contralateral limbs. The patient was pain-free with a full ROM at the left hip and there were no abnormal findings on radiologic examination during the 9-month follow up. Case 2 result: The rehabilitation protocol used was similar to case 1, including straight-leg raise exercises and resisted knee extension strengthening exercises. Postoperatively, there was significant relief of pain and no abnormal findings on physical examination of lag test. The patient was symptom-free without any limitation of activity during the 9 months of follow up. (Figure 3B). Case 3 result: The standardized postoperative rehabilitation training program was undertaken as before. The patient was allowed free active and passive ROM on postoperative day 1. Resisted knee-extension strengthening exercises were prohibited for 6 weeks. At the 9-month follow up, the patient had returned to her previous activity level with quadriceps femoris muscle strength 5 out of 5, and without pain.

## Discussion

Calcium hydroxyapatite crystal deposition disease is defined as painful periarticular calcific deposits in tendons and soft tissues. As other periarticular soft tissues may also be involved, calcific tendinitis constitutes a subgroup of the calcium hydroxyapatite crystal deposition disease process [9]. Clinically, the disorder is usually monoarticular and mainly affects persons between 40 and 70 years of age [10]. The presenting symptom is pain aggravated by joint motion. The most frequently reported anatomic region involved in this disease is the shoulder [2]. In the hip area, the calcifications are commonly found in the region of the gluteal tuberosity involving the tendons of the hip abductors or the origin of the vastus lateralis [11]. In contrast, calcific tendinitis of the rectus femoris is a rare disease [5] first recognized in 1967 by King and Vanderpool [12].

The straight head of the rectus femoris originates at the anterior inferior iliac spine and the reflected head at the ilium above the acetabulum [13]. In our three cases, calcific tendinitis developed in the reflected head of the rectus femoris, which surrounds the acetabular rim with a wide insertion largely overlapped by the capsular attachment. Compared to the direct head, calcific tendinitis in the reflected head of the rectus femoris is more likely to produce an acute episode because of the proximity to the hip capsule and ease of clinical detection [14]. An acute pain exacerbation caused by rupture of calcific material [15] into the joint was not presented by our patients, and their symptoms had a slow gradual onset.

According to the literature, periarticular calcific tendinitis can be classified as acute or chronic. In chronic calcific tendinitis, mild or moderate pain lasts for 2 to 24 months [10]. In our patients, a chronic pattern of calcific tendinitis of rectus femoris origin was diagnosed based on the characteristic radiographic appearance and intraoperative picture.

Calcium hydroxyapatite crystal deposition is a multifactorial disease. Repetitive trauma has been cited as a factor, especially in the shoulder region [2]. Recently, tendinous tears of hip abductors were compared to rotator cuff tears in the shoulder [16]. As in case 3, a history of sprain at the affected hip was associated with the patient’s symptoms, which supported the hypothesis that repetitive trauma plays a role in calcification.

The calcium deposit undergoes an evolution (precalcific stage; calcific stage with formative phase, resting period, resorption, and postcalcific stage), which ultimately remodels normal tissue [17]. It is widely accepted as self-limited disease, but some of the refractory patients do need treatment because the duration of symptoms varies from months to years. Conservative treatment [15,18], local injection [19], ultrasonic therapy [20], radiotherapy [12], and open surgical [4] removal are successful in relieving pain and regaining ROM, in which conservative treatment is the first-line therapeutic approach and surgical removal is usually reserved for a small number of patients with long-lasting symptoms not responding to conservative therapy [21,22]. In the three cases reported above, we considered this persistent pain, worsening despite conservative treatment, as an indicator of the chronic type and decided to opt for surgical therapy. In addition, physical examination and radiographic evaluation disclosed calcific tendinitis of the origin of the rectus femoris combined with intra-articular chondropathy and labral tears. Because the arthroscopic approach is a less morbid alternative to classical open surgery, with the advantages of minimal disturbance of tendons and rapid recovery, we recommended surgical therapy with arthroscopic removal as the ideal treatment option for these patients.

Hip arthroscopy has closed the gap between conservative and invasive treatment. However, a sensible indication and exact preoperative evaluation with advanced imaging techniques are essential to achieve optimal final results [23]. Kandemir *et al*. [7] previously showed excellent results with arthroscopic removal of calcific tendinitis of the gluteus medius and minimus tendon at the superolateral margin of the greater trochanter in their case report. Schmitz *et al*. [8] suggested that arthroscopic removal of calcinosis circumscripta between the labrum and capsule in the hip is a good option, especially in young patients with a high activity level and without any symptoms of hip osteoarthritis.

The rectus femoris muscle is an extensor of the leg at the knee and flexor of the leg at the hip. Some concerns about complications were raised by postoperative weakness of the quadriceps femoris muscle. We conclude that arthroscopic removal of the calcific deposits in the reflected head of the rectus femoris can lead to an excellent clinical outcome without compromising the functional integrity of the knee extensor mechanism. This might depend on that, in our series, the anatomic region of calcific deposits was exclusively located within the reflected head of rectus femoris close to the capsule of the hip, whereas the straight head of rectus femoris at the anterior inferior iliac spine were not involved. Another possible reason is that an endoscopic approach was chosen for these patients to avoid dissection through the straight head of rectus femoris in contrast with open surgery. In addition, it is important to emphasize that the longitudinal incision in the capsule fibers and reflected head tendon fibers should be small, partial thickness and a sparing single opening and that a transverse incision should not be performed as this may lead to retraction of tissue preventing the tendon from healing. Probably for the aforementioned reasons, none of our patients complained about limitation of the knee extension and clinical testing of the quadriceps femoris unit revealed a negative lag test with muscle strength scored as 5 out of 5. Even though the clinical relevance of these pathologic changes in the reflected head remains unclear, regarding the excellent functional results, one might suppose that the intact integrity of the straight head may have the prime role in knee extension and minor structural changes of the reflected head may not influence the clinical presentation. Due to the limited number of cases of calcific tendinitis of the rectus femoris tendon, further investigation will be required to assess the long-term effects of arthroscopic treatment.

## Conclusions

In summary, we present three cases of hip arthroscopic removal of calcific tendinitis of the origin of the rectus femoris tendon combined with intra-articular lesions. To the best of our knowledge, no reports exist about arthroscopic treatment for chronically painful calcific tendinitis of the origin of the rectus femoris. Although an uncommon entity, calcific tendinitis should be considered as a part of the differential diagnosis of acute or chronic hip pain. Hip arthroscopic surgery is an effective and promising therapeutic modality for calcium hydroxyapatite crystal deposition disease in the hip joint.

## Abbreviations

AL: Anterolateral; CT: Computer tomography; MRI: Magnetic resonance imaging; PL: Posterolateral; ROM: Range of motion.

## Competing interests

The authors declare that they have no competing interests.

## Authors’ contributions

GC conceived and designed the experiments. YF performed the experiments. LY analyzed the data. XP contributed reagents/materials/analysis tools. XP wrote the manuscript. All authors read and approved the final manuscript.
